# Procyanidin B3 Prevents Articular Cartilage Degeneration and Heterotopic Cartilage Formation in a Mouse Surgical Osteoarthritis Model

**DOI:** 10.1371/journal.pone.0037728

**Published:** 2012-05-22

**Authors:** Hailati Aini, Hiroki Ochi, Munetaka Iwata, Atsushi Okawa, Daisuke Koga, Mutsumi Okazaki, Atsushi Sano, Yoshinori Asou

**Affiliations:** 1 Department of Plastic and Reconstructive Surgery, Tokyo Medical and Dental University, Tokyo, Japan; 2 Department of Internal Medicine, Keio University, Tokyo, Japan; 3 Division of Veterinary Surgery, School of Veterinary Medicine, Nippon Veterinary and Life Science University, Tokyo, Japan; 4 Department of Orthopedic Surgery, Tokyo Medical and Dental University, Tokyo, Japan; 5 Research and Development Division, Kikkoman Corporation, Chiba, Japan; University of Western Ontario, Canada

## Abstract

Osteoarthritis (OA) is a common disease in the elderly due to an imbalance in cartilage degradation and synthesis. Heterotopic ossification (HO) occurs when ectopic masses of endochondral bone form within the soft tissues around the joints and is triggered by inflammation of the soft tissues. Procyanidin B3 (B3) is a procyanidin dimer that is widely studied due to its high abundance in the human diet and antioxidant activity. Here, we evaluated the role of B3 isolated from grape seeds in the maintenance of chondrocytes *in vitro* and *in vivo*. We observed that B3 inhibited H_2_O_2_-induced apoptosis in primary chondrocytes, suppressed H_2_O_2_- or IL-1ß−induced nitric oxide synthase (iNOS) production, and prevented IL-1ß−induced suppression of chondrocyte differentiation marker gene expression in primary chondrocytes. Moreover, B3 treatment enhanced the early differentiation of ATDC5 cells. To examine whether B3 prevents cartilage destruction *in vivo*, OA was surgically induced in C57BL/6J mice followed by oral administration of B3 or vehicle control. Daily oral B3 administration protected articular cartilage from OA and prevented chondrocyte apoptosis in surgically-induced OA joints. Furthermore, B3 administration prevented heterotopic cartilage formation near the surgical region. iNOS protein expression was enhanced in the synovial tissues and the pseudocapsule around the surgical region in OA mice fed a control diet, but was reduced in mice that received B3. Together, these data indicated that in the OA model, B3 prevented OA progression and heterotopic cartilage formation, at least in a part through the suppression of iNOS. These results support the potential therapeutic benefits of B3 for treatment of human OA and heterotopic ossification.

## Introduction

Osteoarthritis (OA) is a common disease in the elderly due to an imbalance in cartilage degradation and synthesis. In OA, articular chondrocytes appear to be eliminated by apoptosis [1]–[3]. The number of apoptotic cells in articular cartilage is significantly higher in OA patients than in healthy subjects. In response to cytokine stimulation, articular chondrocytes can produce a variety of reactive oxygen species (ROS), including peroxynitrite, superoxide anions, nitric oxide (NO), and hydrogen peroxide (H_2_O_2_) [4]–[6]. IL-1β induces chondrocyte death only when used in combination with oxygen radical scavengers or with a CD95 agonist [7], [8]. The apoptosis-enhancing pathway induced by IL-1β depends on the generation of ROS [8]. Primary OA chondrocytes show both spontaneous and inducible levels of lipid peroxidation activity [9]. Thus, ROS are among the key inflammatory mediators involved in chondrocyte apoptosis observed in OA. H_2_O_2_ induces apoptosis in many cell types and may mediate cartilage degeneration associated with inflammatory joint diseases that induce chondrocyte apoptosis [10], [11].

NO has been increasingly recognized as a signaling intermediate of IL-1−induced responses in many cell types [4], [12], including chondrocytes [13], [14]. NO also regulates aggrecanase activity and induces aggrecan degeneration in chondrocytes [15]. Endogenously synthesized NO reduces cartilage proteoglycan synthesis in response to cytokines such as IL-1 [13]. IL-1 plays a pivotal role in the pathophysiology of OA by inducing a cascade of inflammatory and catabolic events, including the synthesis of prostaglandin E_2_ (PGE_2_) and NO. IL-1 also alters chondrocyte anabolism by suppressing the synthesis of extracellular matrix (ECM) components, such as proteoglycan and type II collagen, and by enhancing the production of matrix metalloproteinases (MMPs) [13].

Heterotopic ossification (HO) occurs when ectopic masses of endochondral and intramembranous bone form within the soft tissue and muscle around joints, in subcutaneous tissues, and in ligaments [16]. HO that occurs around joints can result in pain, loss of motion, and impaired function. A number of recent studies have shown that there is an increased rate of HO in patients with serious injuries and head trauma [17]. A safe and effective primary prophylaxis for HO is under clinical investigation [18].

Grape seed proanthocyanidins (GSP) are powerful antioxidant polyphenols [19], [20], and various epidemiologic and *in vivo*/*in vitro* experimental studies [21]–[23] have suggested that proanthocyanidins derived from fruits, vegetables, and beverages might decrease the risk of several lifestyle diseases. Recent studies have shown that proanthocyanidins have various therapeutic properties, such as radical scavenging, and exhibit a number of health benefits, including antiulcer, antiallergy, antidental caries, and antitumor activity. In addition, proanthocyanidins may inhibit food allergies, activate hair follicle growth, and protect cells from ultraviolet radiation [24]–[30].

Proanthocyanidins are obtained from many kinds of plants as a complex mixture of structurally related components. The structural diversity of polyphenols makes it difficult to determine the biological properties of individual components. The absorption of polyphenols also depends on their molecular weight. Because of their large molecular weight, proanthocyanidin polymers are likely not as easily absorbed by the small intestine. A major portion of ingested polyphenols (75–99%) is not detected in the urine, whereas procyanidin dimers are detected in the serum of rats and humans after GSP ingestion [31], [32]. Procyanidin B3 (B3) is a procyanidin dimer that is widely studied due to its abundance in the human diet [33]–[35]. B3 and other procyanidin dimers can be absorbed by the small intestine [36] and have relevant antioxidant activities [37]. In the present study, we evaluated the role of B3 isolated from grape seed extracts in the differentiation and survival of chondrocytes *in vitro*. Furthermore, the potential ability of B3 to protect articular cartilage *in vivo* and prevent HO was estimated using a surgically-induced osteoarthritis model.

## Materials and Methods

### Reagents

B3 was isolated in the laboratory from proanthocyanidin-rich grape seed extracts, which contained 82% proanthocyanidins, provided by Kikkoman Co. (Chiba, Japan). Isolation was carried out according to the method by Zhao *et al.* [33]. Briefly, B3 was purified using reverse and normal phase HPLC. The isolated B3 was characterized by NMR and HPLC/MS, and the purity was determined to be 97% by HPLC/UV.

### Animals

C57BL/6J mice were obtained from Sankyo Labo Service (Tokyo, Japan), and fed under standard conditions with food and water *ad libitum*. All of the animal experiments were approved by the Animal Care and Use Committee of Tokyo Medical and Dental University.

### Cell Culture Conditions

The mouse chondrogenic ATDC5 cell line was obtained from the RIKEN cell bank (Tsukuba, Japan). Cells were maintained in DMEM/F12 (1∶1) medium containing 5% FCS, 10 µg/ml human transferrin (Invitrogen A/S, Tastrup, Denmark), and 3×10^−8^ M sodium selenite (Sigma-Aldrich, Copenhagen, Denmark) at 37°C in a humidified atmosphere containing 5% CO_2_. Chondrogenic differentiation of ATDC5 cells was performed as previously described [38]. Briefly, ATDC5 cells were seeded at a density of 6×10^3^×cells/cm^2^ in 6-well or 24-well plates and cultured for 4 days. When cells became confluent, the medium was replaced with fresh medium supplemented with insulin (10 µg/ml).

Primary epiphyseal chondrocytes were isolated from 5-day-old mice as previously reported [39]. Briefly, cartilage tissues, including the femoral heads, femoral condyles and tibial plateau, were cut into small pieces and digested twice for 45 min each with 3 mg/ml type I collagenase. Then, the cartilage pieces were incubated in 0.5 mg/ml type I collagenase at 37°C in a thermal incubator with 5% CO_2_ overnight. The next day, cell aggregates were dispersed via pipetting. The cells were cultured in 12-well plates with 5×10^4^ cells per well in DMEM/F12 medium containing 10% FBS and antibiotics.

### RNA Extraction and Real-time RT-PCR

Total RNA was extracted from chondrocytes and cell lines using TRIzol according to the manufacturer’s directions. Real-time PCR was performed using the SuperScript III Platinum Two-Step qRT-PCR Kit with SYBR Green on the Mx3000P® QPCR System. Briefly, 0.5 µg total RNA was mixed with 10 µl 2× RT reaction mix and 2 µl RT, and then incubated for 50 min at 42°C. The reaction was terminated by heating for 5 min at 85°C. The cDNA mixture was then incubated for 30 min at 37°C in the presence of RNase H. The PCR reaction was carried out using a mixture of Platinum SYBR Green qRT-PCR Super-Mix UDG, the template cDNA, 10 mM of the primer mix, and DNase-free H_2_O with a total volume of 20 µl per well. The cycling conditions were performed as indicated in the Invitrogen SuperScript™ III Platinum two-step qRT-PCR kit with SYBR Green. Gene expression was normalized to the endogenous control GAPDH, and fold changes in the genes of interest were determined using the comparative threshold cycle (Ct) method [40]. The qRT-PCR primers are listed in Table 1.

**Table 1:** Primers for real-time PCR.

	Forward 5̀-3̀	Reverse 5̀-3̀
Gapdh	ACTCACGGCAAATTCAACGGC	ATCACAAACATGGGGGCATCG
Aggrecan	ATCAAGTGGAGCCGTGTTTC	CTGGGGATGTCGCATAAAAG
iNOS	GGATTTCAAAGACCTCTGGATC	ATACTTTATGCCACCAACAATGG
Col1a1	CACCCTCAAGAGCCTGAGTC	AGACGGCTGAGTAGGGAACA
Col2a1	CTGGCTGGCATCGTTAC	AGAGTGGTTCCCCTGGTGAG
Col10a1	ACCAGGAATGCCTTGTTCTC	ATGCTGAACGGGACCAAACG

### Measurement of Cellular Injury

Primary chondrocytes were incubated at 37°C in 96-well plates. After 2 days of culture, the medium was changed to 100 µl DMEM/F12 supplemented with 10% FBS. Subsequently, the cells were treated with H_2_O_2_, H_2_O_2_+B3 or Tween 20, as a positive control. After 24 h of culture, cellular injury was quantitated by measuring lactate dehydrogenase (LDH) release using an LDH Cytotoxicity Assay Kit (WAKO, Tokyo, Japan).

**Figure 1:**
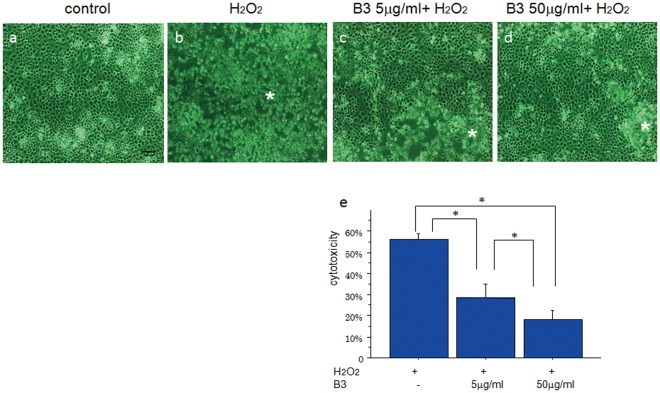
Phase contrast microscopy images of primary chondrocytes incubated with 500 µM H_2_O_2_ and increasing concentrations of B3 or vehicle control. The majority of H_2_O_2_ -treated cells were already detached and floating (b, asterisk), whereas control and B3-treated cells had started rounding but mostly remained adherent (c, d, asterisk). Scale bar  = 200 µm. Cell viability of chondrocytes treated with H_2_O_2_ and B3 as determined by the LDH assay (e). B3 prevented H_2_O_2_-induced chondrocyte apoptosis in a dose-dependent manner. Values represent the mean and SD; n = 5 samples/group. *p<0.05.

### Surgical Induction of OA

Male mice (3 months old) were divided into two groups: B3 and control (n = 10 each). While the mice were under general anesthesia, a medial capsular incision was made and the left knee joint exposed. The medial collateral ligament was transected, and the medial meniscus was removed using a surgical microscope with a microsurgical technique, as previously reported [41]. The right knee underwent a sham operation. All of the animals were allowed unrestricted activity and were provided food and water *ad libitum*. None of the mice died during the experimental period. Five days after surgery, B3 or the vehicle control was administered orally (1 mg/10 g body weight) once a day.

### Assessment of OA Severity

Whole knee joints were removed by dissection, fixed in 4% paraformaldehyde, and decalcified in EDTA. After dehydration and paraffin embedding, 5-µm frontal serial sections were cut from the whole knee joint. Two sections were obtained at 100-µm intervals and then stained with Safranin O–fast green and haematoxylin and eosin. The OA severity in the tibial plateau was evaluated according to Mankin’s histologic grading system [42], [43].

**Figure 2:**
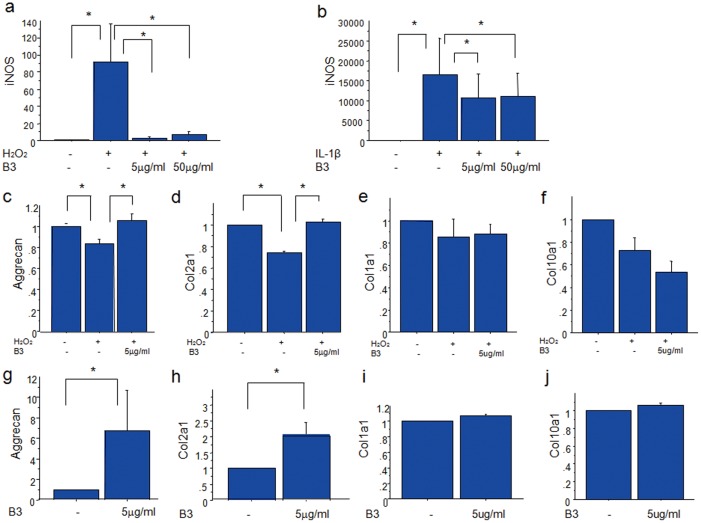
Effects of B3 on primary chondrocytes and ATDC5 cells. (a, b) B3 prevents H_2_O_2_- (a) or IL-1β(b)-induced iNOS synthesis. Primary chondrocytes were stimulated with 50 µM H_2_O_2_ in the absence or presence of increasing concentrations of B3 for 24 h. iNOS synthesis was assessed by real-time RT-PCR. Each column represents the mean ± SD of five separate experiments. *p<0.05. (c−f) B3 prevents H_2_O_2_-induced reduction of proteoglycan (c) and Col2a1 (d) synthesis. Primary chondrocytes were stimulated with 50 µM H_2_O_2_ in the absence or presence of 5 µg/ml B3 for 24 h. mRNA expression of proteoglycan (c), Col2a1 (d), Col1a1 (e) and Col10a1 (f) was assessed by real-time RT-PCR. Results represent the mean ± SEM of three independent experiments. *p<0.05 (g−j) B3 enhances aggrecan and Col2a1 synthesis in ATDC5 cells. ATDC5 cells were incubated in DMEM/F12 with 5%FBS. After the cells reached confluency, they were treated with differentiation medium. After 4 days of culture, the cells were treated with B3 or the vehicle control for 24 h. mRNA expression of aggrecan (g), Col2a1 (h), Col1a1 (i) and Col10a1 (j) were determined by real-timeRT-PCR. The results represent the mean ± SEM from three independent experiments. *p<0.05.

### TUNEL Assay

The TUNEL assay was performed using a TUNEL detection kit according to the manufacturer’s instructions (Takara Shuzo, Kyoto, Japan). Briefly, knee joint sections were incubated with 15 µg/ml of proteinase K for 15 min at room temperature and then washed with PBS. The sections were immersed in TdT Enzyme diluted with Labeling Safe Buffer (provided in the kit) and then incubated for 90 min at 37°C in a humid atmosphere. After washing in PBS, the slides were examined by fluorescence microscopy.

### Immunohistochemistry

iNOS expression was examined by immunohistochemistry with anti-mouse iNOS antibody used according to the manufacturer’s instructions (Abcam Biochemicals, Cambridge, UK). Briefly, tissue sections were incubated overnight at 4°C with a rabbit polyclonal anti-mouse iNOS antibody, followed by a 30-min incubation at room temperature with a biotinylated goat anti-rabbit IgG antibody. Next, the signal was visualized using peroxidase-conjugated avidin and diaminobenzidine from a Vectastain kit, according to the manufacturer’s instructions (Vector Laboratories, Burlingame, CA, USA).

### Statistical Analysis

Data are expressed as the mean ± SD. Statistical analysis was performed with the Mann−Whitney U test or Bonferroni/Dunn test. *p* values <0.05 were considered significant.

**Figure 3:**
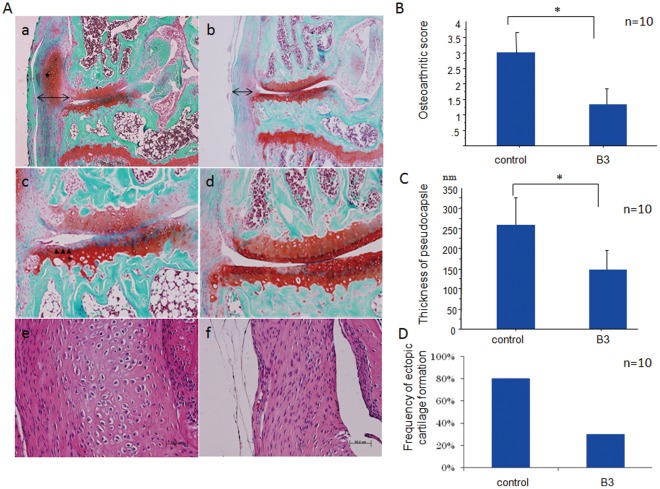
Histological analysis of surgically-induced osteoarthritis (OA) in the knee joints of mice after administering B3 or the vehicle control. OA was surgically induced in 12-week-old mice, and the knee joints were harvested 4 weeks later. B3 or the vehicle alone (control) was orally administered 5 times a week beginning 5 days after surgery and continuing until the end of the experiment (n = 10). A, Representative histologic features (a−d, Safranin O staining; e and f, H&E staining) of the knee joints of control (a, c, e) and B3-treated (b, d, f) mice. Degenerative changes in the articular cartilage were ameliorated in B3-treated mice (b, d) as compared to controls (a, c). Note the thicker medial pseudocapsule in the control group (a, b, arrows), and the ectopic cartilage formation in the pseudocapsule (a, asterisk). e, f, High magnification images of the pseudocapsules. Note that the control pseudocapsule is occupied with hypertrophic chondrocyte-like cells (e), whereas only thin fibrous cells are observed in B3-treated mice (f). B, Histological scoring of cartilage destruction according to Mankin’s score, * *p*<0.05. C, The thickness of the pseudocapsule was significantly reduced in the B3-treated group. Values represent the mean ± SD of 10 samples per group. *p<0.05. D, The incidence of ectopic cartilage formation at the medial pseudocapsule was reduced by B3 treatment.

## Results

### Effects of B3 on H_2_O_2_-induced Chondrocyte Apoptosis

The protective effects of B3 against H_2_O_2_-mediated chondrocyte cell death were evaluated in epiphyseal primary chondrocytes. Cells were pre-incubated with increasing concentrations (5 and 50 µg/ml) of B3, and then treated with 500 µM H_2_O_2_. Treatment with H_2_O_2_ induced apoptosis in approximately 60% of the cells after 24 h of treatment. However, pre-incubating the cells with 5 and 50 µg/ml B3 significantly reduced the extent of apoptosis in a dose-dependent manner (Figure 1). The concentrations of B3 were determined according to the results of a pilot study. Anti-apoptotic effects of B3 were observed from around 5 µg/ml to 1 µg/ml.

### Effects of B3 on iNOS mRNA Expression in Chondrocytes

Primary chondrocytes were stimulated with 50 µM H_2_O_2_ in the absence or presence of increasing concentrations of B3, and iNOS production was evaluated by real-time RT-PCR. B3 treatment suppressed H_2_O_2_-induced iNOS production (Figure 2a). Similarly, iNOS production stimulated by 10 ng/ml IL-1β was also suppressed in the presence of B3 (Figure 2b). An LDH assay demonstrated that this inhibition was not due to reduced cell viability (data not shown).

### Effects of B3 on Chondrocyte ECM Synthesis

To investigate the effects of B3 on H_2_O_2_-induced reduction of chondrocyte differentiation marker gene expression, primary chondrocytes were stimulated with H_2_O_2_ in the absence or presence of increasing concentrations of B3 for 24 h. B3 treatment inhibited the H_2_O_2_-induced decrease in mRNA expression of chondrocyte differentiation markers, including aggrecan and Col2a1 (Figure 2c and d). By contrast, the expression of the chondrocyte hypertrophy markers Col1a1 and Col10a1 was not affected by B3 treatment (Figure 2e and f).

Next, we evaluated the effects of B3 on chondrocyte differentiation using ATDC5 cells. ATDC5 cells were grown to confluency and then cultured in differentiation medium. After 4 days, the cells were treated with B3 for 24 h. mRNA synthesis of aggrecan and Col2a1, was significantly enhanced by B3 treatment (Figure 2g and h), while Col1a1 and Col10a1 expression was not affected (Figure 2i and j).

**Figure 4:**
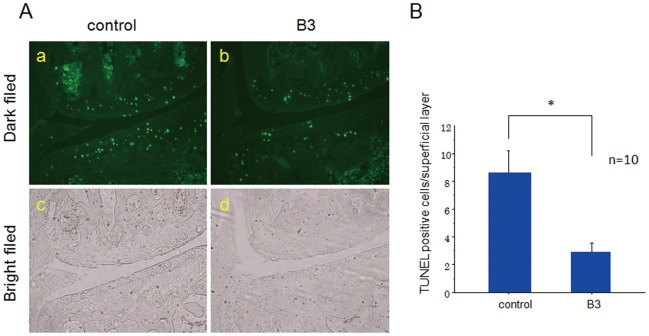
A. TUNEL staining of OA cartilage sections. TUNEL staining was examined by dark-field (a and b) and bright-field (c and d) microscopy. The number of TUNEL-positive cells was increased in the superficial layer of joint cartilage in OA control mice (a), but was significantly reduced in B3-treated mice (b). B, The number of TUNEL-positive cells in the superficial layer of articular cartilage as determined by fluorescence microscopy. Values represent the mean ± SD of 10 mice per group. *p<0.05 (Mann−Whitney U test).

### Prevention of Cartilage Destruction and Ectopic Cartilage Formation by B3 Administration in a Surgically-induced OA Model

To examine whether B3 prevents cartilage destruction, OA was surgically induced in C57BL/6J mice, then B3 or a vehicle control was orally administered. OA was induced by performing a medial collateral ligament transection and medial meniscectomy on the left knees; sham operations were performed on the right knees. B3 or the vehicle control was administered orally 5 days/week beginning 5 days after the operation, and mice were euthanized 4 weeks after the operation. Histological examination revealed that B3 administration markedly protected the articular cartilage from proteoglycan depletion and prevented alterations in surface structure (Figure 3Aa−d). The Mankin’s histologic OA grading score in B3-treated animals was ∼50% of the values in the control group (*p*<0.05, Figure 3B).

In this OA model, the medial meniscus and medial collateral ligament are surgically removed, followed by formation of a pseudocapsule, which is composed of fibrous tissues. In control OA mice, ectopic cartilaginous tissues were frequently observed in the thick pseudocapsule as a result of chronic inflammation (Figure 3Aa and e). By contrast, the thickness of the pseudocapsule was markedly reduced in B3-treated mice along with a reduced frequency of ectopic cartilage formation (Figure 3Ab and f, C and D). These observations suggested that B3 suppressed chronic inflammation in the surgical region.

Chondrocyte apoptosis is increased in OA cartilage [2], [44]. These observations prompted us to investigate the effects of B3 administration on chondrocyte apoptosis. TUNEL-positive cells were abundant among chondrocytes in the superficial layer of articular cartilage in control mice with surgically-induced OA, whereas TUNEL-positive cells were rarely observed in B3-treated mice (Figure 4Aa and b). The number of TUNEL-positive chondrocytes at the superficial layer of the joint cartilage in the B3-treated group was almost one-third of that observed in the control group (*p*<0.05, Figure 4B). These data indicated that the antiapoptotic effects of B3 protected the articular cartilage.

**Figure 5:**
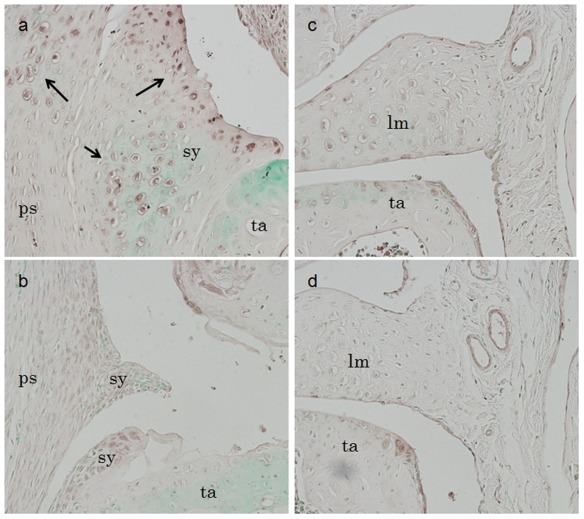
iNOS expression in a murine OA model. iNOS protein expression was enhanced in the synovial tissues and the pseudocapsule around the surgical region in control mice (a, arrows), but was reduced in animals receiving B3 (b). iNOS expression and background levels were similar at the lateral region of each knee joint in B3-treated (d) and control mice (c). sy, synovium; ps, pseudocapsule; lm, lateral meniscus; ta, tibial articular cartilage.

To examine if B3 supplementation prevented cartilage destruction by inhibiting iNOS expression, immunohistological analysis for iNOS was performed. iNOS protein expression was enhanced in the synovial tissues and the pseudocapsule around the surgical region in control mice (Figure 5a, arrows), whereas its expression was reduced by B3 administration (Figure 5b). These data indicated that B3 prevented aberrant articular cartilage degeneration and heterotopic cartilage formation, at least in a part, through suppression of iNOS in the OA model.

## Discussion

Chondrocyte apoptosis has been implicated in the pathogenesis of degenerative joint diseases, including osteoarthritis and rheumatoid arthritis [1], [2], [10]. H_2_O_2_ is both exogenously supplied and endogenously produced in rheumatoid arthritis [1], and it can induce chondrocyte apoptosis [10]. Thus, we used H_2_O_2_-treated chondrocytes as an *in vitro* model to examine the ability of B3 to prevent chondrocyte apoptosis. Indeed, B3 blocked H_2_O_2_-induced chondrocyte apoptosis *in vitro*.

In this study, both H_2_O_2_ and the inflammatory cytokine IL-1ß induced iNOS mRNA expression in chondrocytes, and B3 significantly suppressed iNOS mRNA expression arising from both stimuli (Figure 2). The iNOS and NO levels in the synovial ﬂuid and serum of patients with osteoarthritis are higher than those in healthy individuals [45], [46]. iNOS is expressed following stimulation with a variety of inflammatory agents, such as endotoxins or cytokines [47], and leads to the production of NO in inﬂammatory settings.

NO induces oxidative stress in chondrocytes, and as a result, enhances aggrecan degradation and chondrocyte apoptosis [3], [11]. NO also attenuates the synthesis of cartilage matrix proteins [48]–[50]. In our study, H_2_O_2_ reduced mRNA synthesis of aggrecan and Col2a1, which are cartilage differentiation markers, while B3 significantly prevented these negative effects.

In this study, B3 administration markedly prevented OA progression in articular cartilage. Reduced progression of experimental osteoarthritis is observed *in vivo* upon the selective inhibition of iNOS [51]. Furthermore, selective inhibition of iNOS reduces the progression of experimental osteoarthritis *in vivo* [52]. In a collagen-induced arthritis model, joint pathology is significantly inhibited in NOS-deficient mice [13]. These findings are consistent with our observation that orally administered B3 prevents chondrocyte degeneration via regulating iNOS synthesis, as shown in our surgically-induced OA model. Although B3-mediated iNOS inhibition may be one means by which B3 prevents OA, the molecular target of B3 is still unknown.

Furthermore, TUNEL staining revealed that B3 suppressed chondrocyte apoptosis in a mouse OA model. Many studies have shown that apoptotic cell death occurs at an increased rate in osteoarthritic cartilage, detrimentally impacting articular cartilage maintenance. Combined with the *in vitro* data, this observation may indicate that B3 had both antioxidant and anti-inflammatory effects.

We also demonstrated that B3 treatment prevented heterotopic cartilage formation near the surgical region, but enhanced the differentiation of chondrocyte-like ATDC5 cells *in vitro*. This discrepancy may be due to the effects of B3 on abnormal inflammation at the pseudocapsule. In this study, ectopic cartilage formation was triggered by the trauma of knee surgery, *e.g.,* resection of the meniscus and medial collateral ligament, and a fibrous pseudocapsule was formed. In this location, a cascade of chronic inflammation likely occurred as a result of joint instability. In a typical cascade, iNOS is first expressed following stimulation with a variety of inflammatory agents, such as endotoxins or cytokines [47]. Then, NO promotes inflammation by enhancing the production of inflammatory cytokines [53] and prostaglandins [54]. The latter are implicated in the formation of heterotopic bone [55]. On a related note, anthocyanins inhibit NF-κB activation via TNF-α, resulting in the inhibition of VEGF expression [56]. The NF-κB pathway is involved in regulating prostaglandin expression via COX-2 activation [57], [58]. Thus, B3 may prevent heterotopic ossification by inhibiting prostaglandin activity through suppression of iNOS synthesis and inactivation of the NF-κB pathway.

GSP have several bioactivities. They limit adipogenesis and function as insulinomimetic, anti-inflammatory, and antioxidant agents as shown in this study [32]. Dimeric and trimeric oligomers are the most powerful procyanidin molecules that most closely mimic complete GSP [32]. Grape seed extracts might be expected to have same efficiency because they include several procyanidin dimers and trimers in addition to B3, but additional experiments will be required to confirm this hypothesis.

In conclusion, we have demonstrated that B3 prevented cartilage destruction in an experimental murine model of OA and heterotopic cartilage formation. Our results suggest that B3 prevented chondrocyte apoptosis by directly affecting chondrocytes *in vivo*. These results support the potential therapeutic applications of B3 in humans with OA and HO.
